# On the feasibility of improved target coverage without compromising organs at risk using online adaptive stereotactic partial breast irradiation (A‐SPBI)

**DOI:** 10.1002/acm2.13813

**Published:** 2022-11-09

**Authors:** Steven K. Montalvo, Nathan Kim, Chika Nwachukwu, Prasanna Alluri, David Parsons, Mu‐Han Lin, Bin Cai, Tingliang Zhuang, Brian Hrycushko, Liyuan Chen, Robert Timmerman, Asal Rahimi

**Affiliations:** ^1^ Department of Radiation Oncology UT Southwestern Harold C. Simmons Cancer Center Dallas Texas USA

**Keywords:** adaptive, partial breast irradiation, stereotactic

## Abstract

**Purpose:**

Describe an early‐adopting institution's experience with online adaptive radiation for stereotactic partial breast irradiation.

**Methods and materials:**

Retrospective review of 22 women treated between May 2021 and March 2022 with adaptive stereotactic partial breast irradiation. A total of 106 of 110 fractions were evaluated for dosimetric changes in target coverage and organ‐at‐risk (OAR) dose. Patient set up with stereotactic wooden frame and adapted per fraction. Treatment and planning times were collected prospectively by radiation therapists.

**Results:**

Scheduled PTV_30 Gy_ was <95% in 72.1% and <90% in 38.5% of fractions, and both PTV and CTV coverage were improved significantly after adaption, and 83.7% of fractions were delivered as adapted per physician choice. There was no difference in OAR coverage. Average adaptive treatment planning took 15 min and average time‐on‐couch was 34.4 min.

**Conclusions:**

Adaptive stereotactic breast irradiation resulted in improved target coverage with equivalent dosing to OARs in an efficient and tolerated treatment time. Improved target coverage allowed for decreased PTV margins compared to prior trial protocols that may improve acute and late toxicities.

## 1 INTRODUCTION

Significant efforts in the last several decades have been spent reducing the toxicity of treatment for women with low‐risk breast cancers. Postoperative whole‐breast irradiation remains an important treatment for women who elect for a lumpectomy; however, in carefully selected patients, accelerated partial breast irradiation (APBI) has been shown to be a safe and effective alternative treatment^1^, ^2^, ^3^, ^4^ and ASTRO has published an evidence‐based consensus statement guiding its use.^5^ Our institution has used stereotactic guidance in APBI and has shown favorable toxicity and cosmetic outcomes by negating planning target volume (PTV) expansions due to fiducial‐guided real‐time image tracking and respiratory gating.^6^, ^7^, ^8^

Online adaptive radiation therapy (ART) promises a new wave of image‐guided radiation therapy that modifies beam delivery to account for geometric deformations of patients’ anatomy. ART improves delivery accuracy and precision of radiation prescription to targets that can change from time of simulation to treatment day.^9^ Women with breast cancer who qualify for APBI are ideal candidates for ART. Lumpectomy cavity changes occur during radiation treatment and the breast setup can be variable resulting in large inter‐fraction movement of the target that has been compensated by large PTV expansions.^10^ We hypothesize that ART can decrease the volume of irradiated tissue, either by adapting to gross tumor volume (GTV) reduction (e.g., seroma resorption during the course of treatment) or eliminating added PTV margin to account for inter‐fraction motion, thereby reducing toxicity from radiation similar to other stereotactic partial breast irradiation platforms.^11^ Herein, we report on the dosimetric benefits and the efficiency of delivery among patients receiving adaptive stereotactic partial breast irradiation (A‐SPBI) within the last year at our department by reducing the PTV margins compared to standard 3D‐conformal APBI plans.

## 2 METHODS

**FIGURE 1:**
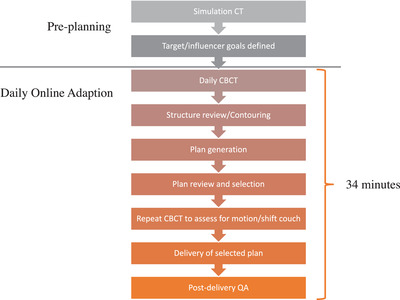
Flow diagram demonstrating stepwise process of adaptive planning. Grayed background indicates pretreatment planning, whereas orange background demonstrates procedure on treatment day. Average couch‐to‐door time was 34 min.

We retrospectively reviewed 22 patients who received A‐SPBI at our institution between May 2021 and March 2022. All patients received 30 Gy in 5 fractions with a total of 110 fractions. Six fractions were excluded from analysis due to initial setup error or due to lack of an adapted plan for comparison, resulting in 104 fractions and 208 paired plans available for analysis. ART was performed on a commercially available ART unit (Ethos, Varian Medical Systems, Inc., Palo Alto, CA, USA). We followed our previously published treatment planning objectives and wooden frame patient setup; however, gold fiducial markers were not placed.^7^ The pretreatment plan was designed using an intensity‐modulated radiation therapy technique. Each plan used 9–11 fields designed based on the beams‐eye‐view to avoid primary radiation transmission through the contralateral breast and heart. The beam arrangement and the optimization goals set in the initial plan were utilized for online ART. The workflow prior to delivery included a simulation visit followed by treatment planning, where targets and organs at risk (OARs) are contoured and “influencer” structures identified. “Influencer” structures are those that may change over time and influence the shape of the target, such as lung, heart, and breast contours. These structures were auto‐contoured then manually reviewed and adjusted on days of treatment prior to treatment delivery. On treatment days, the following sequence of events occurred: daily cone‐beam CT (CBCT), reviewing influencers, recontouring/reviewing OARs and targets on CBCT, plan generation, plan review and selection, repeat CBCT to assess for motion during planning and apply couch shifts, delivery of selected plan, and postdelivery quality assurance (Figure 1). “Scheduled” plans were defined as the pretreatment plan, so called initial reference plan, overlaid on the CBCT with dose calculated based on the anatomy and contours from the CBCT. “Adapted” plans were plans re‐optimized to anatomy and contours from the CBCT. Simulation CTs were available at time of adaptive contouring to help ensure accurate contouring.

PTV and clinical target volume (CTV) volumes, PTV volume receiving 30 Gy (*V*_30 Gy_), CTV *V*_30 Gy_, ipsilateral breast receiving 50% of the prescription dose (*V*_50%_) and *V*_100%_, skin *D*_max_ (0.03 cm^3^), ipsilateral lung *V*_9 Gy_, and heart *V*_150 cGy_ (if left‐sided disease) were collected for both the scheduled and adapted plans for each fraction. CTV volume was created from GTV, defined as cavity delineated by surgical clips, plus a 10 mm radial margin limited from musculature of chest wall and 5 mm from skin. Skin was defined as a 5 mm inner margin from the body contour. PTV was a 0–5 mm radial expansion of CTV volume per physician discretion. Time for adaptive planning was collected prospectively by radiation therapists for 87 fractions. Paired *t*‐test analysis was used for parametric data and Wilcoxon rank sum test was used for nonparametric data. Analyses were done in R v4.2.1 (www.r‐project.org). This study was approved by our Institutional Review Board.

## 3 RESULTS

The mean patient age was 64.9 years (range 42–80). All patients met ASTRO APBI “suitable” or “cautionary” category. Eighteen patients had pathologic T1‐2N0M0 invasive ductal carcinoma and 4 patients had ductal carcinoma in situ (1/4 grade 2 and 3/4 grade 3), and 11 patients had left‐sided breast cancer. Overall, 83.7% (87/104) of fractions were delivered adapted as per physicians’ discretion after review. CTV and PTV volumes ranged from 15.7 to 197.3 cm^3^ (mean 78.3 cm^3^) and 33.4–226.6 cm^3^ (mean 116.7 cm^3^), respectively. The scheduled PTV *V*_30 Gy_ was <95% in 75 fractions (72.1%) and <90% in 40 fractions (38.5%). For adapted fractions, the PTV *V*_30 Gy_ and CTV *V*_30 Gy_ coverage were significantly improved compared to scheduled plans, median (IQR) 91.2 (84.3%–93.9%) versus 95% (95%–95%) and 97.2 (93.4%–99.2%) versus 99.6% (98.9%–99.8%), respectively, *p* < 0.0001 (Table 1). There were no statistical differences in PTV or CTV coverage when scheduled plans were chosen over adapted plans. Ipsilateral breast dose, ipsilateral lung dose, skin dose, and heart dose (for left‐sided disease) were not statistically different between adapted plans and scheduled plans. Contouring and plan generation took on average 9.7 ± 4.9 and 5.3 ± 2.5 min, respectively, whereas overall average time‐on‐couch was 34.4 ± 9.0 min, and all treatment sessions were fully completed by patients.

**TABLE 1:** Comparison of targets and organ‐at‐risk (OAR) coverage between scheduled versus adapted plans on adapted or non‐adapted fractions

	Median (%)	Interquartile range (%)	*p* Value
PTV *V*_30 Gy_ adapted			
Scheduled	91.2	84.3–93.9	<0.0001
Adapted	95	95–95	
PTV *V*_30 Gy_ non‐adapted			
Scheduled	95.5	94.3–97.1	0.0975
Adapted	95	95–95	
CTV *V*_30 Gy_ adapted			
Scheduled	97.2	93.4–99.2	<0.0001
Adapted	99.6	98.9–99.8	
CTV *V*_30 Gy_ non‐adapted			
Scheduled	99.6	97.1–99.8	0.3056
Adapted	99.7	99.4–99.9	
Heart *V*_150 cGy_ adapted			
Scheduled	0.7	0–8.0	0.3044
Adapted	0.7	0–5.4	
Breast *V*_50%_ adapted			
Scheduled	26.4	22.3–32.1	0.8413
Adapted	27.8	22.2–31.8	
Breast *V*_100%_ adapted			
Scheduled	10	8.1–12.3	0.6789
Adapted	10	8.4–13.0	
Lung *V*_9 Gy_ adapted			
Scheduled	6	3.4–8.2	0.4553
Adapted	5.8	2.5–7.4	
Skin $D_{0.03cm^{3}}$ per fraction, adapted	Median (cGy)	Interquartile range (cGy)	
Scheduled	634	618.8–647.0	0.4915
Adapted	634	619.8–641.0	

## 4 DISCUSSION

Our data demonstrate that online ART significantly improved the coverage of the CTV and PTV in patients who received A‐SPBI, and that adapted plans were more likely to be chosen over scheduled plans by the treating physicians. Though follow‐up is short, A‐SPBI may be superior to conventional APBI for disease control by reducing marginal misses of the CTV/PTV by accounting for inter‐fraction shifts and/or deformations of lumpectomy cavities. Importantly, online ART allows for significant PTV margin reduction by eliminating inter‐fraction uncertainty. The RAPID trial and University of Florence studies both defined PTVs as a 1 cm expansions from CTV accounting for positional variability of the lumpectomy cavity, as a geographic miss could result in a poor clinical outcome in these highly curable patients.^2^, ^4^ Online ART obviates the need for such large PTV expansions as there is higher certainty in target localization and thus dose delivery. In our series, we most commonly limited PTV expansions to 3–5 mm; however, in one case of a large cavity, a PTV margin of 0 mm was used. With further research and follow‐up, PTV expansions may be further limited to 1–2 mm (depending on tumor location) to account for intra‐fractional motion only, which may further reduce acute and late toxicities and improve cosmesis.^8^, ^11^, ^12^ During commission of our two Ethos systems, the CyberKnife end‐to‐end head‐and‐neck phantom was digitally deformed and preplanned in Ethos for ART. The real phantom was this treated using the Ethos ART workflow, of CBCT follow by deformation of the planning CT and contours, ending with optimization of the ART plan on the synthetic CT. Across both machines, the mean targeting error recorded was 0.54 ± 0.08 mm (0.47–0.68 mm) which indicates limiting margins is feasible on this platform. In the setting of lumpectomy seromas, A‐SPBI may reduce the risk of target under‐coverage or overtreatment by adapting to changing cavities. Indeed, one patient in this series had a seroma that steadily decreased in size over treatment such that her GTV decreased from 14.39 to 7.76 cm^3^ between simulation and her fifth adapted fraction, resulting in a PTV that was 27% smaller than initially planned. Adaptation and preventing potential target misses become even more important when single‐fraction SPBI is under investigation. Further, A‐SPBI is delivered via intensity modulation, which has more favorable cosmetic outcomes compared to 3D‐conformal APBI as evident from worsened cosmetic outcomes in RAPID trial, possibly related to the large PTV margins used on protocol.^3^, ^4^, ^13^, ^14^, ^15^

ART was first proposed in 1997 and has been incorporated into radiation treatments in both offline and online forms.^9^, ^16^ Recently, proliferation of commercially available MRI‐guided and CBCT‐based online ART technology has piqued interest in the radiation oncology community. The CBCT‐based Ethos by Varian has simplified the process of online ART such that adaptation can occur within typical treatment times.^17^, ^18^ Our experience shows that A‐SPBI planning takes less than 15 min and mean start‐to‐finish treatment time of 34 min per fraction is comparable to a conventional 3D‐conformal APBI treatment time. Further, no patients who started A‐SPBI failed to complete their course as intended and, in our experience, tolerated treatment setups and length of treatment well.

A‐SPBI has some disadvantages using the current version of Ethos. Plan generation must be optimized to efficiently create target volumes. Once initial planning objectives and priorities are applied to online ART plan optimization, there is no opportunity for modification of the plan. The physician and planner must actively consider the deformation of the relevant anatomy and its potential impact on target coverage and OAR sparing in the preplanning phase. For example, if the GTV is near the skin surface and yet an aggressive skin OAR constraint was chosen, then the system may prioritize the skin constraint during adaptation over target coverage, resulting in a suboptimal plan. It is important to communicate with the planner to select appropriate influencer templates, automate volume creations (e.g., CTV expansions), and select achievable constraints to optimize workflow and plan quality.

In our institution, we use CyberKnife (Accuray Inc., Sunnyvale, CA), GammaPod (Xcision Medical Systems, LLC, Columbia, MD), and Ethos for S‐PBI. On preliminary comparison of plans using these technologies, low‐dose spread appears worse in A‐SPBI compared to other stereotactic PBI platforms, such as CyberKnife or GammaPod, or what is expected from brachytherapy. However, A‐SPBI has some advantages over these technologies. For example, A‐SPBI obviates the need for indwelling catheters used in brachytherapy, permanent fiducial markers used with CyberKnife, and is not limited by tumor location or breast size that occasionally poses challenges with GammaPod. Further, A‐SPBI delivery time is comparable to CyberKnife. An additional drawback of A‐SPBI is the significant resources ART requires. ART requires physicians and physicists to be at the console for contouring, plan evaluation, and plan selection which, at our center, took on average 15 min per fraction. Although this does take physician and physicist time at the console, there may be unmeasurable time‐savings associated with improved toxicity profile of a more focused treatment.

## 5 CONCLUSIONS

Our experience shows A‐SPBI is a novel treatment delivery approach that is efficient, well tolerated, and has the potential to improve clinical outcomes by reducing margins, delivering intensity modulated beams, and adapting to deformable targets. More prospective clinical studies are needed to validate the potential benefits of A‐SPBI at an early‐adopting institution, such as ours.

## AUTHOR CONTRIBUTIONS

Steven K. Montalvo primarily acquired and analyzed data and drafted this work. Asal Rahimi primarily designed the study and oversaw data analysis and drafting of the manuscript. Nathan Kim, Chika Nwachukwu, Prasanna Alluri, David Parsons, Mu‐Han Lin, Bin Cai, Tingliang Zhuang, Brian Hrycushko, Liyuan Chen, and Robert Timmerman each contributed to the design, acquisition, analysis, and interpretation of the data, assisted in the drafting and revising of this work, and approved the final draft for publication.

## CONFLICTS OF INTEREST

The authors declare that there is no conflict of interest.

## Data Availability

Research data are stored in an institutional repository and will be shared upon request to the corresponding author.

## References

[1] Vaidya JS , Bulsara M , Baum M , et al. Long term survival and local control outcomes from single dose targeted intraoperative radiotherapy during lumpectomy (TARGIT‐IORT) for early breast cancer: tARGIT‐A randomised clinical trial. BMJ. 2020;370:m2836. 32816842 10.1136/bmj.m2836 PMC7500441

[2] Meattini I , Marrazzo L , Saieva C , et al. Accelerated partial‐breast irradiation compared with whole‐breast irradiation for early breast cancer: long‐term results of the Randomized Phase III APBI‐IMRT‐Florence Trial. J Clin Oncol. 2020;38:4175‐4183. 32840419 10.1200/JCO.20.00650

[3] Vicini F , Shah C , Tendulkar R , et al. Accelerated partial breast irradiation: an update on published Level I evidence. Brachytherapy. 2016;15:607‐615. 27475478 10.1016/j.brachy.2016.06.007

[4] Whelan TJ , Julian JA , Berrang TS , et al. External beam accelerated partial breast irradiation versus whole breast irradiation after breast conserving surgery in women with ductal carcinoma in situ and node‐negative breast cancer (RAPID): a randomised controlled trial. Lancet. 2019;394:2165‐2172. 31813635 10.1016/S0140-6736(19)32515-2

[5] Correa C , Harris EE , Leonardi MC , et al. Accelerated Partial Breast Irradiation: executive summary for the update of an ASTRO evidence‐based consensus statement. Pract Radiat Oncol. 2017;7:73‐79. 27866865 10.1016/j.prro.2016.09.007

[6] Rahimi A , Morgan HE , Kim DW , et al. Cosmetic outcomes of a phase 1 dose escalation study of 5‐fraction stereotactic partial breast irradiation for early stage breast cancer. Int J Radiat Oncol Biol Phys. 2021;110:772‐782. 33476737 10.1016/j.ijrobp.2021.01.015

[7] Rahimi A , Thomas K , Spangler A , et al. Preliminary results of a phase 1 dose‐escalation trial for early‐stage breast cancer using 5‐fraction stereotactic body radiation therapy for partial‐breast irradiation. Int J Radiat Oncol Biol Phys. 2017;98:196‐205. 28586960 10.1016/j.ijrobp.2017.01.020

[8] Rahimi AS , Kim DN , Leitch M , et al. Early follow‐up of multi‐institutional trial of phase I dose escalation using single fraction stereotactic partial breast irradiation (S‐PBI) for early‐stage breast cancer. Int J Radiat Oncol Biol Phys. 2021;111:e228. 10.1016/j.ijrobp.2021.10.010 34710523

[9] Lim‐Reinders S , Keller BM , Al‐Ward S , et al. Online adaptive radiation therapy. Int J Radiat Oncol Biol Phys. 2017;99:994‐1003. 28916139 10.1016/j.ijrobp.2017.04.023

[10] Jacobson G , Betts V , Smith B . Change in volume of lumpectomy cavity during external‐beam irradiation of the intact breast. Int J Radiat Oncol Biol Phys. 2006;65:1161‐1164. 16682143 10.1016/j.ijrobp.2006.02.009

[11] Hepel JT , Tokita M , MacAusland SG , et al. Toxicity of three‐dimensional conformal radiotherapy for accelerated partial breast irradiation. Int J Radiat Oncol Biol Phys. 2009;75:1290‐1296. 19395195 10.1016/j.ijrobp.2009.01.009

[12] Acharya S , Fischer‐Valuck BW , Mazur TR , et al. Magnetic resonance image guided radiation therapy for external beam accelerated partial‐breast irradiation: evaluation of delivered dose and intrafractional cavity motion. Int J Radiat Oncol Biol Phys. 2016;96:785‐792. 27788951 10.1016/j.ijrobp.2016.08.006

[13] Mukesh MB , Barnett GC , Wilkinson JS , et al. Randomized controlled trial of intensity‐modulated radiotherapy for early breast cancer: 5‐year results confirm superior overall cosmesis. J Clin Oncol. 2013;31:4488‐4495. 24043742 10.1200/JCO.2013.49.7842

[14] Mészáros N , Major T , Stelczer G , et al. Accelerated partial breast irradiation with 3‐dimensional conformal and image‐guided intensity‐modulated radiotherapy following breast conserving surgery – 7‐year results of a phase II trial. Breast. 2020;54:222‐228. 33161336 10.1016/j.breast.2020.10.010 PMC7648201

[15] Lei RY , Leonard CE , Howell KT , et al. Four‐year clinical update from a prospective trial of accelerated partial breast intensity‐modulated radiotherapy (APBIMRT). Breast Cancer Res Treat. 2013;140:119‐133. 23824363 10.1007/s10549-013-2623-x PMC3706719

[16] Yan D , Vicini F , Wong J , et al. Adaptive radiation therapy. Phys Med Biol. 1997;42:123‐132. 9015813 10.1088/0031-9155/42/1/008

[17] Archambault Y , Boylan C , Bullock D , et al. Making on‐line adaptive radiotherapy possible using artificial intelligence and machine learning for efficient daily re‐planning. Med Phys Intl J. 2020;8:77‐86.

[18] Yock AD , Ahmed M , Ayala‐Peacock D , et al. Initial analysis of the dosimetric benefit and clinical resource cost of CBCT‐based online adaptive radiotherapy for patients with cancers of the cervix or rectum. J Appl Clin Med Phys. 2021;22:210‐221. 10.1002/acm2.13425 PMC8504593 34529332

